# Supplementary material for: A Theory of How Columns in the Neocortex Enable Learning the Structure of the World
Source: Front Neural Circuits. 2017 Oct 25;11:81. doi: 10.3389/fncir.2017.00081 (PMC5661005; doi:10.3389/fncir.2017.00081)
Supplement: Supplementary file 2 [file DataSheet1.PDF]

*Supplementary Material*

# A Theory of How Columns in the Neocortex Enable Learning the Structure of the World

**Jeff Hawkins'[1*], Subutai Ahmad[1], Yuwei Cui[1]**

[1] Numenta, Inc, Redwood City, California, United States of America

# Capacity of the HTM Sensorimotor Network

## 1 Overview

This technical note describes capacity analysis of the sensorimotor network. We focus on the following questions.

- How many distinct features and locations can the input layer represent?
- How many (feature, location) pairs can an object contain?
- How many objects can the sensorimotor network store?
- How much should an output cell sample from the input cells?

The first question is related to how diverse the set of sensory features or object locations can be. The second question is related to how complex can individual objects can be. The analysis suggests (1) there is large encoding power in the input layer to represent a diverse set of features and locations; (2) the sensorimotor network allows each object to contain hundreds of distinct (feature, location) pairs; (3) the sensorimotor network can store hundreds of simple objects, where each object contains 10 distinct (feature, location) pairs and (4) output cells can connect to a small fraction of the active input cells with reasonable error rate.

## 2 Capacity of the input layer

In this section we analyze how many sensory features and object locations can be stored in the input layer. In the following analysis, we assume the input layer contains $N$ minicolumns and $M$ cells per minicolumn. At any time, $w$ minicolumns contain active cells, and only one cell becomes active in these $w$ minicolumns. As discussed in the main text, we typically have $N$=150, $M$=16 and $w$=10.

### 2.1 Capacity for sensory features

We assume each sensory feature corresponds to a distinct sparse distributed representation (SDR). The number of unique features that can be represented is

$$\binom{N}{w}$$

In practice, we also want to make sure that distinct sensory features are encoded as SDRs with sufficient differences. This will both ensure a certain amount of noise robustness and reduce the chance of false positive errors in object recognition (see below). Specifically, for each sensory feature, we assume any SDR with more than $\theta$ bits overlap with the template SDR $\mathbf{x}$ is considered as the same sensory feature. Using the concept of SDR overlap set (Ahmad & Hawkins 2016), the number of such SDRs is

$$\sum_{b=\theta}^{w} |\Omega_{\mathbf{x}}(N, w, b| = \sum_{b=\theta}^{w} \binom{w}{b} \times \binom{N-w}{w-b}$$

The capacity for sensory features will reduce in proportion to the size of SDR overlap set. The resulting sensory feature capacity is

$$Feature\ Capacity := \frac{\binom{N}{w}}{\sum_{b=\theta}^{w} \binom{w}{b} \times \binom{N-w}{w-b}}$$

Despite this reduction due to inexact matches, the feature capacity is still a astronomically large number. For $\theta$ =7, the number of unique sensory features is greater than $10^7$. This is much smaller than $\binom{N}{w}$ $(> 10^{15})$ but is more than enough for any practical application.

## 2.2 Capacity for object locations

The same sensory feature can be sensed at different locations. The location information for a given sensory feature is represented by depolarization of individual cells within the minicolumns. Since there are $M$ cells per minicolumn, we can represent each sensory feature at $M^w$ or $16^{10}$ different locations with typical parameters. Again, we might want to consider inexact matches. That is, given the same sensory feature at a learned location and at a random location, if the SDR for the random location shares more than $\theta$ bits with the SDR for the learned location, it will be considered as the same location. The number of SDRs that has inexact (>$\theta$ bits) match with a given SDR is

$$\sum_{b=\theta}^{w} \binom{w}{b} \times M^{w-b}$$

After considering inexact matches, the capacity for locations is

$$Location\ Capacity := \frac{M^w}{\sum_{b=\theta}^{w} \binom{w}{b} \times M^{w-b}}$$

This number is also very large for typical parameters. For $\theta$ =7, *w*=10, *M*=16, the location capacity is more than $10^6$.

These results suggest a very large number of distinct sensory features and locations can be represented in the input layer, such that two different feature/location SDRs have less than $\theta$ bits overlap. Because of this large capacity, we could use a simple random algorithm to assign random SDRs for a set of features or locations. The chance of a "bad" assignment is simply the inverse of the capacity.

We have verified this result by generating 10,000 pairs of random input SDRs. The maximum overlap we observed during simulation is only 3 bits (out of 10 active bits), and the mean overlap across two random input SDRs is only 0.043. It is extremely unlikely for two random SDRs in the input layer to have significant overlap.

## 3 Capacity of the output layer

In the sensorimotor network, each object is represented as a collection of (sensory feature, location) pairs it the input layer, and a stable SDR in the output layer. This requires output cells to form connections to multiple input cells that represent the corresponding sensory features and locations. Therefore, the large number of converging connections from the input layer to the output layer could cause false match errors. That is, an object SDR could be activated by an input SDR that does not correspond to any of the (feature, location) pair of the learned object, but rather because that input SDR also happens to match enough of the connections. Intuitively, such "false match" errors will increase as a function of the number of feedforward connections converging onto each output cell, which is proportional to the number of (feature, location) pairs in each object.

Assuming that each object contains *K* (feature, location) pairs, the pooling step involves connecting to *K* SDRs in the input layer. We assume that *c* active cells are connected for each of the SDR ($c \leq w$). In this section, we first compute how many input cells will be connected to an output cell, and then compute the probability that a random SDR will falsely activate an output cell (false match error).

### 3.1 Number of feedforward connections

In this section we calculate the expected number of feedforward connections. Given an input SDR that represents a (feature, location) pair, the probability that an input cell is active in this SDR, and is subsequently connected to an output cell is

$$p = \frac{w}{NM}\frac{c}{w} = \frac{c}{NM}$$

The probability that an input cell is not selected as a connected cell in any of the *K* SDRs is $(1-p)^K$. Denote the set of input cells that is connected to an output cell that represents this object as $U_{cell}$. The expected number of connected input cells is

$$|U_{cell}| = \left|\bigcup_{i=1}^{K} \mathbf{x}_i\right| = [1 - (1 - \frac{c}{NM})^K]NM$$

which grows sub-linearly as a function of *K*, and almost linear when *K* is small. That is, the number of connected cells is less than c*K*. The number of connected inputs per output cell, as a function of number of (feature, location) pairs per object is shown in Fig. 1. For example, typical cortical pyramidal neurons have hundreds of proximal synapses. If we assume each output cell receives <500 inputs, each object should have less than 100 (feature, location) pairs. Each (feature, location) pair is represented by an input SDR that has 5 cells connected to the output cells ($c$=5, $w$=10).

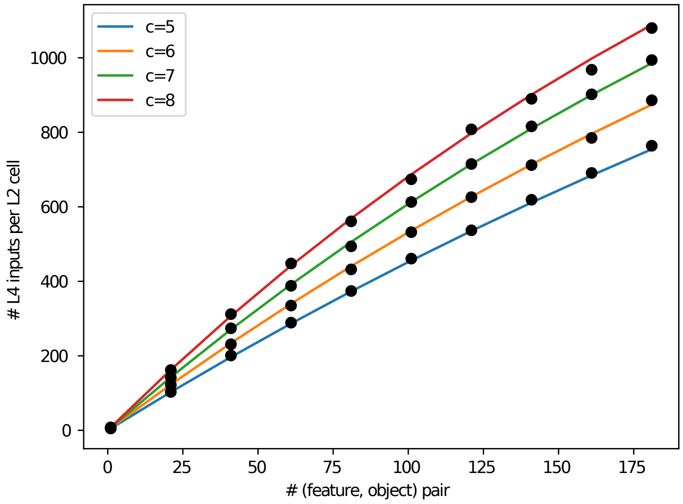


**Figure 1.** Number of connected inputs per output cell, as a function of (feature, location) pairs per object. Colored lines represent theoretical calculations. Black solid dots represent simulation result. In the simulation we created *K* random (feature, location) SDRs. An output cell connects to *c* active bits randomly for each SDR. The number of connections is plotted as a function of *K*.

### 3.2

### 3.3 False match error of a single output cell

In this section we calculate the false match error of a single output cell. We assume that an output cell learns an object with *K* (feature, location) pairs. Consider a new (feature, location) pair that belongs to a different object, if the corresponding input SDR activates the same output cell, we call it a false match error. The chance of a single active bit in this new input SDR belongs to $U_{cell}$ is

$$p_{match}^{bit} = \frac{|U_{cell}|}{NM}$$

The total number of matching bits follows a binomial distribution, that is, given a random input SDR $\mathbf{x}$, the chance of observing $b$ bits matching is

$$P(|\mathbf{x} \cap U_{cell}| = b) = \binom{w}{b} p^b (1-p)^{w-b}$$

where $p = p_{match}^{bit}$.

The cumulative distribution function for binomial distribution is given as

$$F(b;\, w, p) = \sum_{i=0}^{b} \binom{w}{b} p^b (1-p)^{w-b}$$

which is the probability of having less than $b$ bits matching.

The false matching error for one random input SDR, given a matching threshold $\theta$ is

$$P(|\mathbf{x} \cap U_{cell}| > \theta) = 1 - F(\theta;\, w, p)$$

The false match error for a single random input SDR is shown in Fig. 2. The equations are verified by numerical simulations (not shown). The SDR false match error decreases with higher activation threshold (Fig. 2A), and increases with more connections (Fig. 2B). Intuitively, a random SDR is easier to activate an output neuron if there are more connections between the input and output layer and/or if the activation threshold for output cells is low.

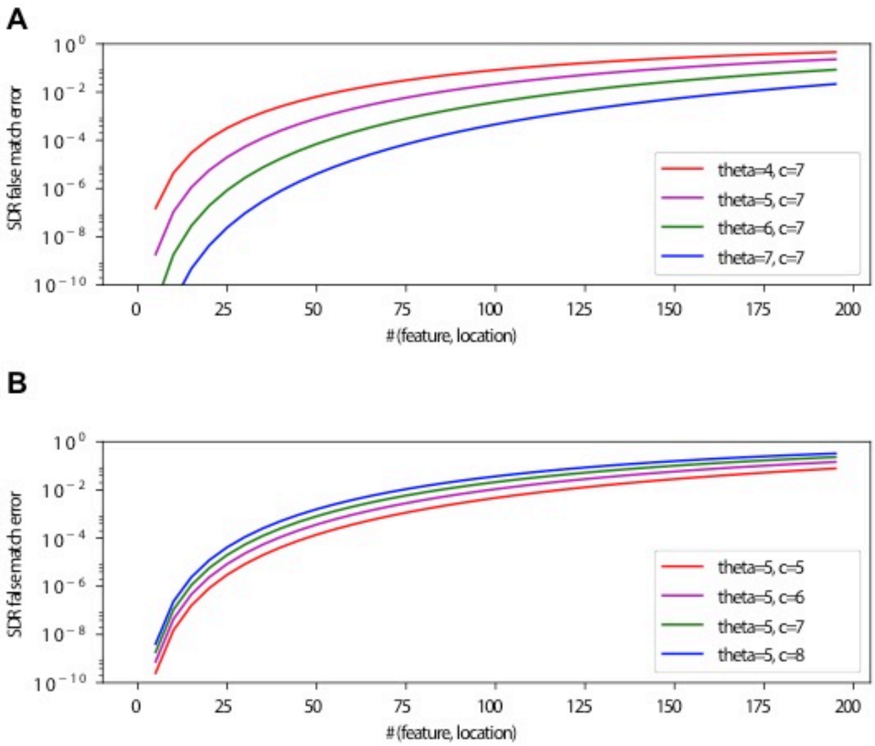


**Figure 2.** SDR false match error as a function of (feature, location) pair number per object. A. Given fixed number of connections, error decreases with higher activation threshold. B. Given fixed activation threshold, error increases with more connections.

Note that in practice it might be tolerable if a small fraction of the input SDRs evoke a different object. Because the sensorimotor algorithm will also integrate over time and across minicolumns, it is very unlikely for several consecutive SDRs to falsely evoke an unwanted object SDR if the individual SDR false match error is low.

### 3.4 False match error of multiple output cells

The false match error derived above is for a single output cell. Typically the output layer will have multiple cells active at any time. For simulations in the main text, we have 40 output cells active for each object. The activations of different output cells that represent the same object are correlated, due to shared feedforward connections, but not identical, due to sampling. If $c$ is large, active output cells are connected to a similar set of input cells, which means if a random SDR falsely activate one output cell, it will also falsely activate most of the other output cells (Fig. 3, left). If there is substantial sampling for each input SDR ($c \ll w$), there will be little overlap among the sets of input cells that are connected to each active output cell (Fig. 3, right). It is very unlikely for a random SDR to falsely activate many output cells simultaneously.

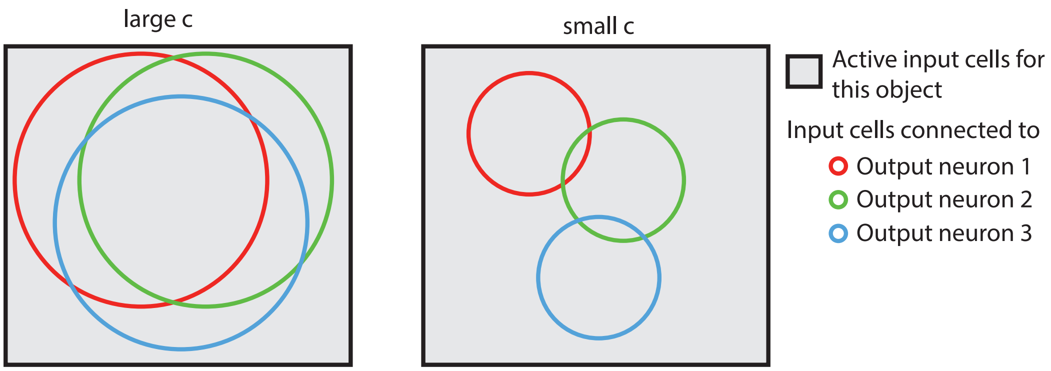


**Figure 3.** L2 connections with large and small connections per input SDR.

Consider a pair of output cells that represent the same object, the expected number of input cells that are connected to both output cells is

$$N_{overlap} = [1 - (1 - \frac{c}{NM}\frac{c}{w})^K]NM$$

The logic behind this equation is similar to that of $|U_{cell}|$. The chance that an input cell is active in one input SDR and is subsequently connected to the first output cell is $c/NM$. Given that it is connected to the first cell, the chance it is also connected to the second output cell is $c/w$. It is obvious that if $c$=$w$, $N_{overlap}$ is the same as $|U_{cell}|$, which means the two output cells have identical feedforward connections.

Given a random input SDR **x**, denote the overlap with the $i$th output cell as

$$o_i(\mathbf{x}) = |\mathbf{x} \cap U_{cell}^i|$$

where $U_{cell}^i$ is the set of input cells that are connected to the $i$th output cell.

Assuming that the $i$th and the $j$th output cell represent the same object. Given a test SDR that has $b_i$ overlap with the $i$th output cell, we would like to compute the chance that it has $b_j$ overlap with the $j$th output cell.

The total number of SDRs with $b_i$ overlap with the $i$th output cell is

$$n_{total} = \binom{|U_{cell}^i|}{b_i}\binom{NM - |U_{cell}^i|}{w - b_i}$$

Among this set of SDRs, the number of the SDRs that also has $b_j$ overlap with the $j$th output cell is

$$n_{qualified} = \sum_{l=minL}^{\text{maxL}} \binom{N_{overlap}}{l}\binom{|U_{cell}^i| - N_{overlap}}{b_i - l}\binom{|U_{cell}^j| - N_{overlap}}{b_j - l}\binom{NM - |U_{cell}^i| - |U_{cell}^j| + N_{overlap}}{w - (b_i + b_j - l)}$$

This equation is a bit complicated, but the intuition is straightforward. We want to count the number of SDRs with $b_i$ overlap with the $i$th output cell and $b_j$ overlap with the $j$th output cell. A qualifying SDR could have $l$ bits connected to both output cells (first term), $b_i - l$ bits connected to the $i$th output cell (second term), $b_j - l$ bits connected to the $j$th output cell (third term), and the rest of $w - (b_i + b_j - l)$ bits are not connected to neither output cells.

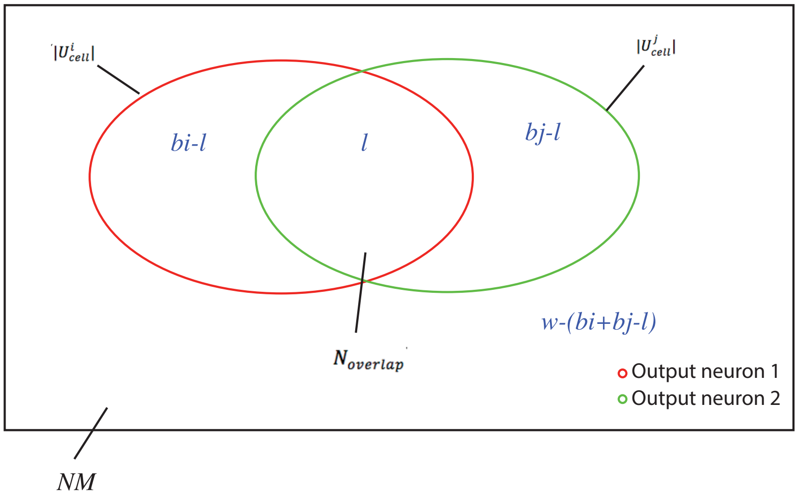


**Figure 4.** Illustration of equation. Black text denotes the size of each compartment. The blue text denotes the number of active bits that lie within each compartment.

The index $l$ is the number of cells that are connected to both SDRs. The maximum possible number of such cells is

$$maxL = \min(b_i, b_j)$$

The minimum number of $l$ is

$$minL = \max(\max(b_i - |U^i_{cell}| + N_{overlap}, 0),$$
$$\max(b_j - |U^j_{cell}| + N_{overlap}, 0),$$
$$\max(b_i + b_j - w, 0)))$$

This is due to a set of non-negative constraints on $b_i - l$, $b_j - l$ and $w - (b_i + b_j - l)$.

Once we have $n_{qualified}$ and $n_{total}$, the desired conditional probability is

$$P[o_j(\mathbf{x}) = b_j | o_i(\mathbf{x}) = b_i] = \frac{n_{qualified}}{n_{total}}$$

Figure 5 shows that this probability decrease quickly as the number of connections per SDR decreases for $o_i$=5 and $o_j$=5. With $c$=5, $K$=40, $N$=150, $M$=16 and $w$=10, the probability is as low as 0.06.

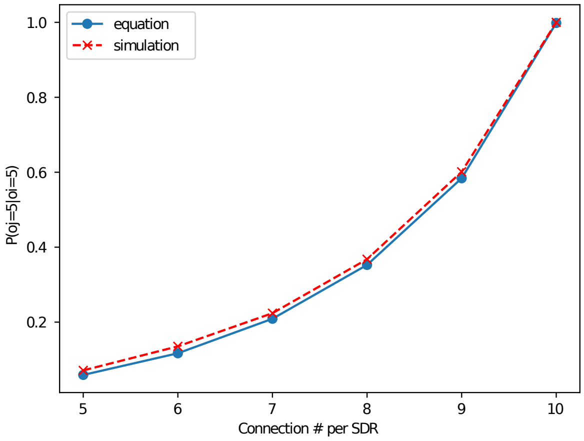


**Figure 5.** Given a SDR with 5 bits overlap with one output cell, the chance that SDR also has 5 bits overlap with a second output cell. The equations agree well with simulation.

The joint probability distribution is

$$P[o_j(\mathbf{x}) = b_j, o_i(\mathbf{x}) = b_i] = P[o_j(\mathbf{x}) = b_j | o_i(\mathbf{x}) = b_i] P[o_i(\mathbf{x}) = b_i]$$

where $P[o_i(\mathbf{x}) = b_i]$ is derived in the previous section.

Given a activation threshold $\theta$, the chance that a random SDR falsely match two output cell is then

$$P^{i,j}_{falsematch} = \sum_{b_i=\theta+1}^{w} \sum_{b_j=\theta+1}^{w} P[o_j(\mathbf{x}) = b_j, o_i(\mathbf{x}) = b_i]$$

We plot the chance of simultaneous false match of two output cells that represent the same object (green curve), together with the false match error of single output cell (blue), as a function of number of connections per input SDR (Fig. 6). The false match error for two output cells is one order of magnitude smaller than that of a single output cell when there is enough sampling (small $c$). This result suggests that by using a distributed representation in the output layer and a small number of connections per input SDR, the false match error can be significantly reduced.

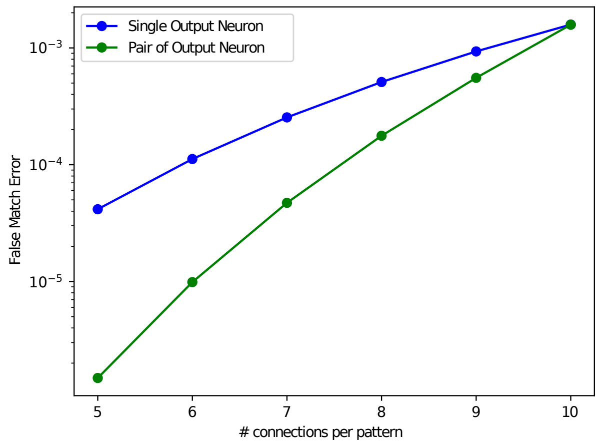


**Figure 6.** False match error for single output cell and pair of output cells, as a function of number of connections per input SDR. The activation threshold is set to 5.

### 3.5 Storage of multiple objects

So far we have discussed the error as a function of complexity of the object in terms of number of (feature, location) pairs per objects. In theory, the false error depends on the number of connections per output cell. Therefore, the network can also store a large number of simple objects. Storing a single large object with 100 (feature, location) pairs uses the same amount of feedforward connections as storing 10 simple objects, each with 10 (feature, location) pairs.

In our capacity simulations, the output layer has a sparsity of 2%. It can thus store at least 50 large objects, each with 100 (feature, location) pairs with very small error rate. The same network can be used to store 500 small objects, each with 10 (feature, location) pairs.

## 4 Simulation

In this section we describe a simulation experiment. We created a set of objects with 100 (feature, location) pairs. The input layer has 150 minicolumns, 16 cells per minicolumn, and 10 active cells at any time. Among these 10 active cells, individual output cells randomly connect to $c$=5 input cells, and has an activation threshold of $\theta$ =4. There are 40 active output cells at any time. We repeated the experiment 10,000 times. On each trial, we computed the number of falsely activated output cells.

With these parameters, individual output cells are connected to 452 input cells on average. The chance of having at least one output cell falsely activated is 0.203, which is quite high. However, it is very unlikely to have a large number of output cells falsely activated simultaneously. The chance of having at least $L$ cells falsely activated decreased rapidly as a function of $L$ (Fig. 7). The chance of simultaneously activating more than half of the output cells (20/40) is as low as 0.004. Therefore, we can still reliably recognize the objects by considering multiple output cells.

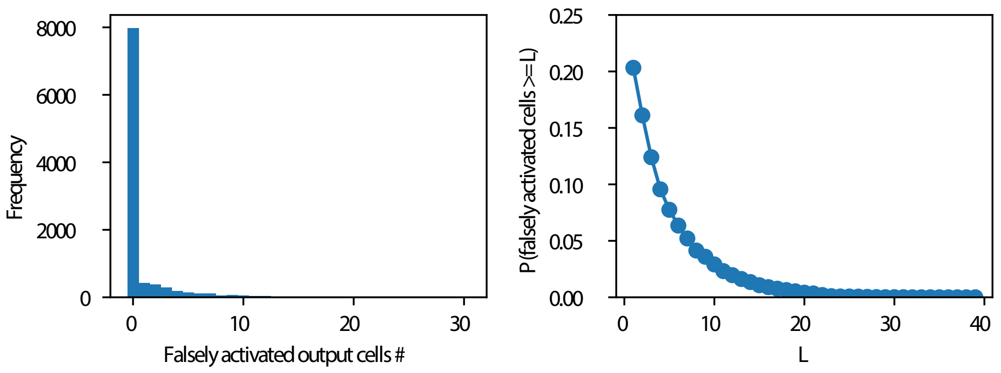


**Figure 7.** Left: Distribution of output cells that are falsely activated simultaneously. Right: The probability of simultaneously activating $L$ output cells decreases rapidly as a function of L.

## 5 Reference

Ahmad S, Hawkins J. 2016. How do neurons operate on sparse distributed representations? A mathematical theory of sparsity, neurons and active dendrites. *arXiv*. 1601.00720 [q-NC]

# Improved Robustness to Noisy and Ambiguous Inputs with Feedback

In this supplemental note we describe an example experiment that demonstrates the importance of the feedback signal when the inputs are noisy and ambiguous. In this experiment, we trained the network on 40 objects. Each object has 10 features and 10 locations. Each feature is randomly chosen from a small library of 10 unique features. The objects share the same set of 10 locations. Encoding of feature and location inputs are described in the Methods section of the main text.

During inference, we provided ambiguous location inputs to the network. At each sensation, a union of three location SDRs was simultaneously presented to the network: the correct location SDR is accompanied by two other distracting locations that are randomly chosen from the 10 locations. We also added 30% noise to this union SDR by inactivating 30% of the active bits and simultaneously activate 30% of the inactive bits.

We compared performance of the HTM sensorimotor network with or without feedback (Fig. 8). Without feedback, each active mini-column contains multiple (~2.5) active cells due to the ambiguous location input. When feedback is enabled, there are many fewer active cells in the input layer (Fig. 8A). This is because when multiple cells in a mini-column are predicted, cells with feedback input inhibit cells without feedback. At the same time, there are many fewer active cells in the output layer (Fig. 8B). The network recognized objects faster and achieved a much higher final recognition accuracy (Fig. 8C).

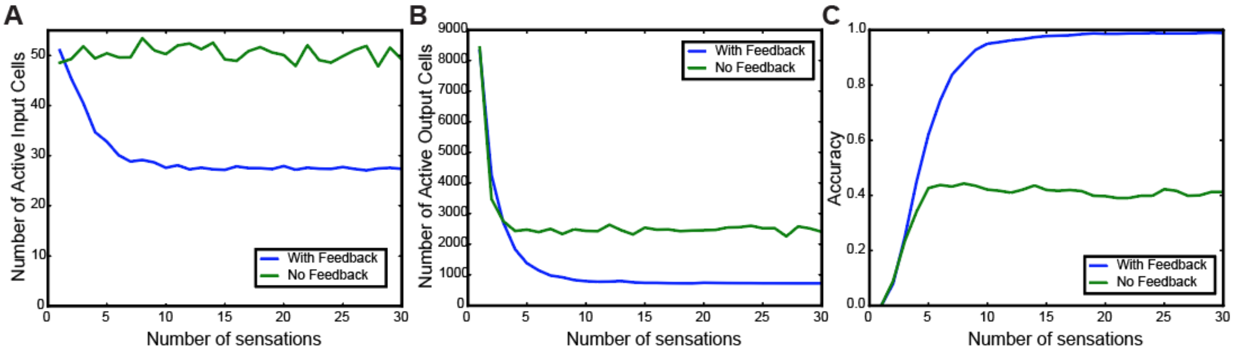


**Figure 8 A.** The number of active input cells vs. number of sensations. **B.** Number of active output cells vs. number of sensations. **C**. The recognition accuracy as a function of the number of sensations. The blue curves represent experiments when feedback is enabled, and the green curves are results when feedback is disabled.

# Ideal Observer and Multiple Column Networks

We compared our multi-column sensorimotor network to an ideal observer model that also observes multiple features per sensation. In this experiment, we trained models on 100 objects. Each object consists of 10 sensory features chosen from a library of 5 to 30 possible features. Each feature is assigned a corresponding location on the object. Note that although each object consists of a unique set of features/locations, any given feature or feature/location is shared across several objects. Encoding of feature and location inputs, and the construction of the ideal observer model are described in the Methods section of the main text.

The graph demonstrates that the behavior of our multi-column sensorimotor network is very close to the non-biological ideal observer model. The network's behavior is slightly worse than ideal when objects are highly confused. (When the pool of unique features is 5, our 4 column network requires an average of 1.7 sensations vs 1.1 for the ideal observer.)

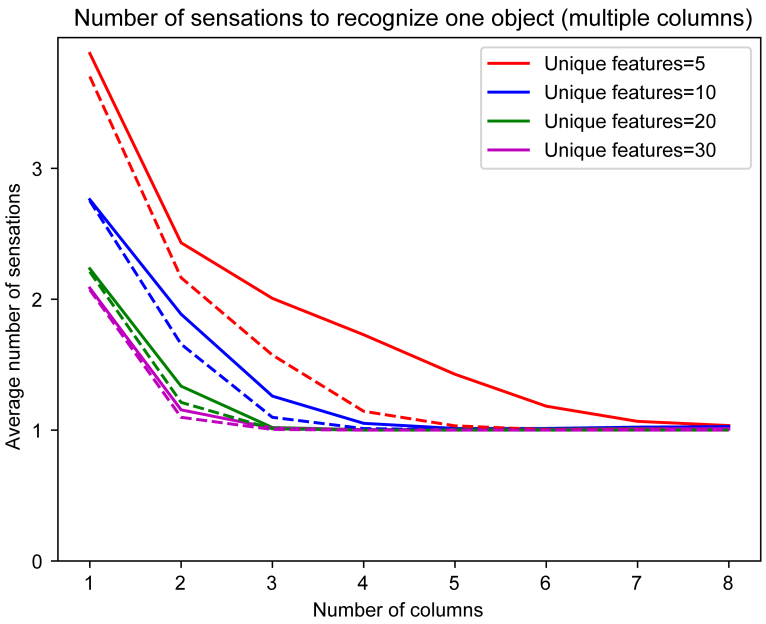


**Figure 9** We plot the mean number of observations needed to unambiguously recognize an object with multi-column networks as the number of columns increases. The required number of sensations rapidly decreases as the number of columns increases, eventually reaching one. The solid curves represent our sensorimotor model. The dashed curves represent the ideal observer model.
